# Antibiotic resistance and molecular characterization of bacteremia *Escherichia coli* isolates from newborns in the United States

**DOI:** 10.1371/journal.pone.0219352

**Published:** 2019-07-05

**Authors:** Bryan K. Cole, Marko Ilikj, Cindy B. McCloskey, Susana Chavez-Bueno

**Affiliations:** 1 Department of Pediatrics, University of Oklahoma Health Sciences Center, Oklahoma City, Oklahoma, United States of America; 2 Department of Pathology, University of Oklahoma Health Sciences Center, Oklahoma City, Oklahoma, United States of America; University of Georgia, UNITED STATES

## Abstract

**Background:**

*Escherichia coli* is a major cause of neonatal sepsis. Contemporary antibiotic resistance data and molecular characterization of neonatal *E*. *coli* bacteremia isolates in the US are limited.

**Methods:**

*E*. *coli* blood isolates, antibiotic susceptibility data, and clinical characteristics were obtained from prospectively identified newborns from 2006 to 2016. The *E*. *coli* isolates were classified using an updated phylogrouping method and multi-locus sequence typing. The presence of several virulence traits was also determined.

**Results:**

Forty-three newborns with *E*. *coli* bacteremia were identified. Mean gestational age was 32.3 (SD±5.4) weeks. Median age was 7 days (interquartile range 0–10). Mortality (28%) occurred exclusively in preterm newborns. Resistance to ampicillin was 67%, to gentamicin was 14%, and to ceftriaxone was 2%; one isolate produced extended-spectrum beta lactamases. Phylogroup B2 predominated. Sequence type (ST) 95 and ST131 prevailed; ST1193 emerged recently. All isolates carried *fimH*, *nlpI*, and *ompA*, and 46% carried the K1 capsule. *E*. *coli* from newborns with bacteremia diagnosed at <72 hours old had more virulence genes compared to *E*. *coli* from newborns ≥ 72 hours old. The *hek/hra* gene was more frequent in isolates from newborns who died than in isolates from survivors.

**Conclusion:**

Antibiotic resistance in *E*. *coli* was prevalent in this large collection of bacteremia isolates from US newborns. Most strains belonged to distinctive extra-intestinal pathogenic *E*. *coil* phylogroups and STs. Further characterization of virulence genes in neonatal *E*. *coli* bacteremia strains is needed in larger numbers and in more geographically diverse areas.

## Introduction

*Escherichia coli* is the most frequent Gram-negative organism that causes neonatal bacteremia. Moreover, *E*. *coli* now surpasses group B *Streptococcus* as a cause of bacteremia in newborns of all gestational ages in several regions of the United States [1–5]. *E*. *coli* is also a major neonatal sepsis pathogen worldwide, particularly in low-income countries [6]. Resistance of neonatal *E*. *coli* invasive isolates in developing countries has been reported to be as high as 100% for ampicillin, and up to 90% for gentamicin [7]. In Spain, 93% of *E*. *coli* isolates causing early-onset sepsis are resistant to ampicillin and 28% are resistant to gentamicin [8]. More worrisome is the presence of extended-spectrum beta-lactamase (ESBL)-producing *E*. *coli* in cases of neonatal bacteremia [9]. ESBL-producing *E*. *coli* has a prevalence as high as 64% in newborns with bacteremia in India [10]. Although some recent studies in the US have documented resistance rates to ampicillin as high as 66–78% in invasive neonatal *E*. *coli* isolates [2, 11], the contemporary prevalence of resistance to various antibiotic classes in neonatal *E*. *coli* bacteremia strains in the United States has not been well defined.

Bacteremia-producing *E*. *coli* strains are distinct from commensal or enteropathogenic strains, and thus are termed extra-intestinal pathogenic *E*. *coli* (ExPEC). The molecular characterization of ExPEC isolates has shed light on the particular genomic traits of these strains, both for epidemiological characterization purposes and to define their virulence properties. Phylogenetic studies have traditionally classified invasive ExPEC strains in phylogroups B2 and D, whereas commensal and diarrheagenic strains are frequently included in phylogroups A and B1 [12]. Furthermore, the pathogenic mechanisms that ExPEC use to produce invasive disease are largely determined by specialized virulence factors (VFs) that predominate in these strains. Phylogroup classification provides general information regarding the source of isolation and the clinical phenotype of diverse *E*. *coli* isolates. However, multi-locus sequence typing (MLST) has the advantage of a more accurate phylogeny classification [13]. Studies describing the molecular epidemiology and the prevalence of genes encoding different VFs in *E*. *coli* strains that produce neonatal bacteremia in the United States are scarce [11].

The objectives of this study were to describe the clinical characteristics of a group of newborns prospectively identified with *E*. *coli* bacteremia and the prevalence of resistance to several antibiotics in the recovered bacterial isolates. We also molecularly characterized the isolates to determine their phylogenetic group, multi-locus sequence type (MLST), and the presence of VFs common to ExPEC strains.

## Materials and methods

### Patients and bacterial isolates collection

This study was approved by the University of Oklahoma Institutional Review Board for the Protection of Human Subjects, IRB#1708. All the clinical records used in this study were anonymised before data were obtained by the researchers. Newborns diagnosed with *E*. *coli* bacteremia were prospectively identified from October 2006 to May 2016 at The Children’s Hospital at OU Medical Center (formerly Children’s Hospital of Oklahoma), a tertiary medical center where approximately 4,500 newborns are delivered every year. The hospital includes a 93-bed neonatal intensive care unit that provides the highest level of neonatal care in the state of Oklahoma. Clinical data and an initial phenotypic and genotypic characterization of the *E*. *coli* isolates obtained from the first 24 newborns included in this study have been published earlier [14]. S1 Table shows the former designation of the initial 24 patients included in the previous publication and the corresponding isolate designation number in the present manuscript.

*E*. *coli* bacteremia was defined as pathogen isolation from a blood culture specimen. The age of bacteremia diagnosis was based on the date and time when the positive blood culture was drawn (not when results became available). Only isolates from each neonate’s first positive blood culture were included in the study. All isolates were maintained in 10% skim milk at -80°C until further analysis. *E*. *coli* identification and antibiotic susceptibility testing were performed in the microbiology clinical laboratory following guidelines by the Clinical and Laboratory Standards Institute according to the methods already described [14]. Multidrug-resistant (MDR) *E*. *coli* was defined as nonsusceptibility to at least 3 antibiotic classes including the following: extended-spectrum cephalosporins, fluoroquinolones, aminoglycosides, carbapenems, and piperacillin or piperacillin/tazobactam [15]. Clinical data and antibiotic susceptibility results were collected from electronic medical records. The Institutional Review Board at the University of Oklahoma Health Sciences Center approved this study; informed consent was waived.

### Phylogroup classification and multi-locus sequence typing (MLST)

The neonatal *E*. *coli* isolates were classified into phylogenetic groups by the updated quadruplex polymerase chain reaction (PCR) method of Clermont et al. [16]. This method has the advantage of allowing a more accurate phylogroup classification of *E*. *coli* compared with its previous version, as it includes strain assignment into phylogroups A, B1, B2, C, D, E, F, and cryptic clade I, as well as cryptic clades II-V. Briefly, genomic DNA from an individual colony was isolated with the DNeasy Blood & Tissue Kit (Qiagen, Germantown, MD). PCR reactions were performed with the following conditions: denaturation 4 min at 94°C, 30 cycles of 5 s at 94°C and 20 s at 59°C (initial quadruplex PCR, and group E if needed), or 57°C (group C, if needed), and a final extension step of 5 min at 72°C. PCR products were stained with ethidium bromide and visualized using agarose gel electrophoresis. Phylogroups were assigned based on the combination of presence or absence of PCR products in each isolate [16].

MLST was determined by amplification and sequencing of seven housekeeping genes (*adk*, *fumC*, *icd*, *pur A*, *gyr B*, *recA*, and *mdh*), as described earlier [14]. Allele identification for each gene was obtained on the https://pubmlst.org/escherichia/ website available from the University of Oxford [17]. The database available at www.enterobase.warwick.ac.uk was used for assigning sequence types (STs) and clonal complexes (CCs) [18].

### Presence of VFs in *E*. *coli* strains

All neonatal *E*. *coli* isolates were additionally tested by PCR for the presence of 12 virulence genes that have been attributed to various pathogenic processes in *E*. *coli* strains associated with extra-intestinal infections, which include neonatal septicemia and meningitis [19–23]. These virulence genes included *cnf1*, cytotoxic necrotizing factor 1; *fimH*, type I fimbriae; *hek/hra*, adhesin/hemagglutinin; *hlyC*, hemolysin; *ibeA*, invasion of brain endothelium A; *iucC*, aerobactin; *iroN*, salmochelin; *kpsMT II*, capsule synthesis; *nlpI*, new lipoprotein I; *ompA*, outer membrane protein A; *papGII-III*, P fimbriae; and *sfa/foc*, S fimbriae. The presence of each virulence factor was determined by PCR using gene specific primers as shown in Table 1 [19–21].

**Table 1:** Primer sequences used for the detection of virulence factors in neonatal *Escherichia coli* bacteremia isolates.

Virulence Gene	Primer sequences (5’-3’)	Product size (base pairs)	Source Reference
*cnf1*	AAGATGGAGTTTCCTATGCAGGAG CATTCAGAGTCCTGCCCTCATTATT	498	Johnson et al. [19]
*fimH*	TGCAGAACGGATAAGCCGTGG GCAGTCACCTGCCCTCCGGTA	508	Johnson et al. [19]
*hek/hra*	CAGAAAACAACCGGTATCAG ACCAAGCATGATGTCATGAC	260	Bingen et al. [20]
*hlyC*	AGGTTCTTGGGCATGTATCCT TTGCTTTGCAGACTGCAGTGT	556	Bingen et al. [20]
*ibeA*	AGGCAGGTGTGCGCCGCGTAC TGGTGCTCCGGCAAACCATGC	170	Johnson et al. [19]
*iroN*	GAAAGCTCTGGTGGACGGTA CGACAGAGGATTACCGGTGT	127	Bonacorsi et al. [21]
*iucC*	AAACCTGGTTTACGCAACTGT ACCCGTCTGCAAATCATGGAT	269	Bingen et al. [20]
*kpsMII*	GCGCATTTGCTGATACTGTTG CATCCAGACGATAAGCATGAGCA	272	Johnson et al. [19]
*nlpI*	TTCGTTGCGACAGCACTTAC TTCCATACGTGCCAGAATCA	120	This study
*ompA*	GAGCCTGGGTGTTTCCTACC TTGTCACAGGTGTTGCCAGT	390	This study
*papGII-III*	CTGTAATTACGGAAGTGATTTCTG ACTATCCGGCTCCGGATAAACCAT	1070	Johnson et al. [19]
*sfa/foc DE*	CTCCGGAGAACTGGGTGCATCTTAC CGGAGGAGTAATTACAAACCTGGCA	410	Johnson et al. [19]

PCR conditions included an initial temperature of 94°C for 5 min, followed by 30 cycles of denaturation at 94°C for 10 s, annealing at 61°C (58°C for *cnf1* and *iroN*) for 20 s, and 72°C for 40 s. Final elongation was at 72°C for 7 min. PCR products were identified by agarose gel electrophoresis and compared to a 100-bp ladder to confirm their appropriate size. Confirmation of the correct amplification of all virulence genes tested with our PCR methods was done by performing sequencing of PCR products in selected recent isolates and verification of sequencing results of each gene by searching the NCBI Microbial Genomes database using the Basic Local Assignment Search Tool (BLAST).

The isolates were also tested for the presence of the K1 capsule with a card latex agglutination test (Wellcogen Remel Europe Ltd, Dartford, Kent, UK). For this test, a single colony of an overnight plate culture of each isolate was tested with the appropriate positive and negative controls according to the manufacturer’s protocol.

### Statistical methods

Statistical analyses were performed with IBM SPSS Statistics v. 24 (IBM Corp, Armonk, NY). Descriptive statistics were used to analyze parametric and nonparametric data, as appropriate. For continuous variables, we used the Shapiro-Wilk test to determine the normality distribution of the data, using a cutoff P value of 0.05.The Fisher exact test was used to compare proportions, and the Student’s t-test or ANOVA were used to compare continuous data, a p value < 0.05 was considered significant. To measure the strength of association between variables, the Pearson Product Moment Correlation, or the Spearman Rank Order Correlation were used as appropriate, and we considered significant a p value < 0.05.

## Results

### Patient demographic and clinical characteristics

Forty-three infants were identified during the study period, of whom 20 (46.5%) were male. Thus, the overall incidence of neonatal *E*. *coli* bacteremia was 0.95 per 1,000 live births for the study period.

Table 2 shows additional demographic and clinical data. The median age of the 43 infants at the time of blood culture sampling was 7 days (interquartile range [IQR] 0–10). Most newborns were premature (n = 33, 77%), while 10 were born at term. The mean gestational age (GA) was 32.3 weeks (SD±5.4). Symptoms prompting the investigation of bacteremia predominantly included an abnormal respiratory effort in 39.5% of patients, and gastrointestinal symptoms in 32.5%. As expected in the neonatal population, even in the case of severe infection most patients were afebrile, and those with fever included only near-term or term newborns in whom this symptom was attributed to the eventual diagnosis of bacteremia. All infants received at full course of parenteral antibiotic treatment for bacteremia, indicating that the clinicians providing medical care made the diagnosis of infection and not contamination in each case.

**Table 2:** Clinical characteristics of newborns with *Escherichia coli* bacteremia.

Collection years	Isolate	Age	GA	Clinical presentation	NEC	Died
2006–2009	SCB 04	7	25	Abdominal distention	yes	no
	SCB 05	10	30	Feeding intolerance	yes	yes
	SCB 09	0	34.6	Asymptomatic	no	no
	SCB 11	9	23.6	Respiratory failure	no	yes
	SCB 12	0	30	Respiratory failure	yes	no
	SCB 13	6	36	Hypoglycemia	yes	yes
	SCB 14	2	25	Respiratory failure	no	yes
	SCB 15	0	40.9	Asymptomatic	no	no
	SCB 17	1	36	Respiratory failure	no	no
2010–2013	SCB 18	8	30	Feeding intolerance	yes	no
	SCB 19	28	40	Fever	no	no
	SCB 20	17	28.6	Abdominal distention	yes	no
	SCB 21	0	26	Respiratory depression	no	yes
	SCB 22	20	29.7	Respiratory failure	no	yes
	SCB 23	6	25	Respiratory failure	yes	yes
	SCB 24	15	31.2	Feeding intolerance	yes	no
	SCB 27	0	31.4	Respiratory depression	no	no
	SCB 28	39	29.2	Feeding intolerance	no	no
	SCB 29	0	29.7	Respiratory depression	no	yes
	SCB 30	17	35.3	Abdominal distention	yes	no
	SCB 31	0	29.3	Respiratory depression	no	yes
	SCB 32	9	24.6	Respiratory depression	no	no
	SCB 33	0	39.9	Respiratory depression	no	no
	SCB 34	6	22.7	Respiratory failure	no	no
	SCB 35	0	27.9	Respiratory depression	no	no
2014–2016	SCB 37	2	41.4	Hypoxemia	no	no
	SCB 38	8	36.6	Fever, lethargy	no	no
	SCB 40	0	31.4	Respiratory depression	no	no
	SCB 41	2	36.9	Abdominal distention, hematochezia	yes	no
	SCB 42	12	34.2	Feeding intolerance	no	no
	SCB 43	9	38	Fever	no	no
	SCB 45	16	35	None recorded	no	no
	SCB 47	0	31	Asymptomatic	no	no
	SCB 49	18	28.4	Feeding intolerance	no	no
	SCB 50	10	38	Fever	no	no
	SCB 52	7	38.7	Fever	no	no
	SCB 54	8	40	Fever	no	no
	SCB 55	7	39.2	Hematochezia, intestinal pneumatosis	yes	no
	SCB 56	29	24.9	Abdominal distention, respiratory failure	yes	yes
	SCB 57	2	33.7	Abdominal distention, hematochezia	yes	yes
	SCB 58	10	39.4	Elevated blood white cell count	no	no
	SCB 59	0	34.2	Respiratory distress, hypotension	no	yes
	SCB 60	5	32.9	hematochezia, pneumatosis	yes	no

Age indicates chronological age in days; GA, gestational age in weeks; NEC, necrotizing enterocolitis;

Additional potential risk factors for the development of bacteremia besides prematurity that were present in this group of newborns are described in S2 Table. Maternal history of chorioamnionitis was present in 53% and meconium-stained fluid was found at birth in 23%. Most infants did not have low 5-minute APGAR scores, a parameter that has been associated to poor outcomes in neonatal sepsis [24]. APGAR scores were assigned using routine criteria (heart rate, respiration, muscle tone, reflexes, color) [25]. Infants were endotracheally intubated and had an indwelling central venous catheter at the time of bacteremia testing in 40% and 42% cases, respectively. Congenital anomalies were present in 25% and predominantly included congenital heart disease or renal abnormalities. Other neonatal conditions were found in 28% of newborns.

Twelve (28%) newborns died. All were born prematurely and had significantly lower mean GA (28.6 weeks, SD±4.1) than those who survived (mean GA 33.6 weeks, ±SD 5.2), p<0.01. None of these infants had any congenital anomalies. The prevalence of other clinical conditions in the group of newborns who died was not significantly different than in those who survived. Eight of the 12 newborns who died (75%) expired within 24 h of blood culture collection, the median interval between blood culture sample and death was 1 day (range 1–10 days). No other infections were found in this group of newborns.

Fourteen (33%) newborns developed NEC, including one term infant. Five of these newborns died. The median age at NEC presentation was 8 days; six newborns presented with NEC within the first week of life.

Cerebrospinal fluid (CSF) was obtained in 14 (32%) newborns, 8 on the same day as the blood culture. CSF from the remaining 6 infants was obtained within 2 days of blood culture collection. Only one CSF culture was positive and yielded *E*. *coli*. The CSF isolate had the same antibiotic susceptibility pattern as the blood isolate, which was K1-positive (SCB38).

### Antibiotic resistance

Table 3 shows the resistance pattern of the neonatal *E*. *coli* isolates to various antibiotic classes along with the antibiotic treatment started immediately upon the suspicion of bacteremia that prompted testing for this disease. Ampicillin resistance was found in a majority of isolates (n = 29, 67%). Six isolates (14%) showed resistance to gentamicin and to tobramycin in addition to ampicillin. All the patients that cleared their bacteremia and survived received antibiotics to which the *E*. *coli* isolate was susceptible to. Three of the newborns infected with isolates resistant to ampicillin/gentamicin/tobramycin died; all three were diagnosed with bacteremia within the first 24 hours of life. Two had received treatment with ampicillin plus gentamicin while awaiting blood culture results, the third one received ampicillin, gentamicin and cefotaxime treatment. The remaining nine patients who died received at least one antibiotic agent that was active against the invasive *E*. *coli* isolate.

**Table 3:** Antibiotic resistance and treatment of neonatal *Escherichia coli* bacteremia isolates with corresponding isolate molecular classification.

Collection years	Isolate	Antibiotic susceptibilities					Antibiotic therapy	PG	MLST	ST complex
		Amp	Gen	Tobra	Cip	T/S				
2006- 2009	SCB 04	S	S	S	S	S	Gen/Van	B2	95	ST95
	SCB 05	R	S	S	S	R	Amp/Gen‡	D	69	ST69
	SCB 09	R	S	S	S	R	Amp/Gen	B2	372	None
	SCB 11	S	S	S	S	S	Amp/Gen‡	B2	141	None
	SCB 12	R	S	S	S	S	Amp/Gen	B2	95	ST95
	SCB 13	S	S	S	S	S	Amp/Gen‡	B2	2830	None
	SCB 14	R	S	S	S	S	Amp/Gen‡	B2	2831	None
	SCB 15	S	S	S	S	S	Amp/Gen	D	501	None
	SCB 17	R	S	S	S	S	Amp/Gen/Clin	B2	2832	None
2010- 2013	SCB 18	R	S	S	R	R	Cefotax/Gen/Clin	B2	131	ST131
	SCB 19	R	S	S	S	S	Amp/Cefotax	B2	95	ST95
	SCB 20	R	S	S	R	S	Clin/Gen	B2	131	ST131
	SCB 21	R	R	I	S	S	Amp/Gen‡	F	117	None
	SCB 22	R	S	S	S	S	Clin/Gen/Oxa‡	B2	131	ST131
	SCB 23	R	S	S	S	R	Clin/Gen‡	B2	131	ST131
	SCB 24	R	S	S	S	R	Clin/Gen	B2	95	ST95
	SCB 27	R	S	S	S	R	Amp/Gen	B2	95	ST95
	SCB 28	S	S	S	S	S	Oxa/Gena	B2	95	ST95
	SCB 29	R	R	R	S	R	Amp/Gen‡	D	69	ST69
	SCB 30	R	S	S	S	R	Vanc/Clin/Gen	D	405	ST405
	SCB 31	R	R	I	S	S	Amp/Gen/Cefotax ‡	B2	131	ST131
	SCB 32	S	S	S	S	S	Amp/Ami	B2	95	ST95
	SCB 33	R	S	S	R	R	Amp/Ami	B2	131	ST131
	SCB 34^#^	R	R	R	R	S	Amp/Ami	B2	131	ST131
	SCB 35*†^#^	R	R	R	R	R	Amp/Ami	D	405	ST405
2014- 2016	SCB 37	R	S	S	R	R	Amp/Ami	B2	1193	ST14
	SCB 38	S	S	S	S	S	Amp/Cefotaxime	B2	95	ST95
	SCB 40	R	S	S	S	S	Amp/Ami	B2	131	ST131
	SCB 41	S	S	S	S	S	Pip- tazo/Amicin	B2	73	ST73
	SCB 42	S	S	S	S	S	Cefotax/Tobra	B2	127	None
	SCB 43	S	S	S	S	S	Amp/Cefotax	B2	95	ST95
	SCB 45	R	S	S	R	R	Amp/Cefotax	B2	1193	ST14
	SCB 47	R	S	S	S	S	Amp/Ami	B2	73	ST73
	SCB 49	R	R	I	S	R	Meropenem	A	10	ST10
	SCB 50	S	S	S	S	R	Amp/Cefotax	B2	7628	ST12
	SCB 52	R	S	S	S	S	Amp/Cefotax	B2	2626	None
	SCB 54	S	S	S	S	S	Amp/Cefotax	B1	1297	None
	SCB 55	R	S	S	S	S	Van/Ami/Metro	B2	95	ST95
	SCB 56	S	S	S	S	S	Van/Ami/Clin‡	B2	127	None
	SCB 57	S	S	S	S	S	Van/Ami/Metro‡	B2	95	ST95
	SCB 58^#^	R	S	R	R	R	Amp/Ami	B2	131	ST131
	SCB 59	R	S	S	S	S	Amp/Ami‡	B2	12	ST12
	SCB 60	R	S	S	R	R	Amp/Ami/Metro	B2	1193	ST14

*Ceftriaxone resistant

† ESBL producer

#Multi-drug resistant isolates

‡ Patient died

Amp indicates ampicillin; Ami, Amicin; Cefotax, cefotaxime; Cip, ciprofloxacin; Clin, Clindamycin; ESBL, extended-spectrum beta-lactamase; Gen, Gentamicin; I, intermediate; Metro, metronidazole; MLST, multi-locus sequence type; Oxa, oxacillin; PG, phylogroup; Pip-tazo, piperacillin-tazobactam; R, resistant; S, susceptible; ST, sequence type; Tobra, tobramycin; T/S, trimethoprim/sulfamethoxazole; Van, vancomycin.

All isolates were susceptible to extended-spectrum cephalosporins with the exception of SCB35, which was resistant to ceftriaxone and was isolated at the end of 2013. This isolate was the only extended-spectrum beta-lactamase (ESBL) producer, and was also resistant to ceftazidime, cefepime, and aztreonam. SCB35 was therefore deemed an MDR strain. Because of the possibility of multiple beta-lactamase producing genes present in this strain, we performed PCR testing to determine the presence of groups I-IV CTX-M beta lactamases in SCB35 with the methods described in S4 Table. PCR amplification and sequencing demonstrated the presence of *bla*_CTX-M-15_ in this isolate. Because the presence of blaCTX-M-15 has been associated to carriage of blaOXA-1 [26], we sought to determine whether SCB35 also carries this beta-lactamase gene. As shown in S4 Table, we demonstrated the presence of blaOXA-1 as well. We also sought the presence of TEM and SHV beta-lactamase genes with PCR methods as indicated in S4 Table. These assays did not demonstrate the presence of these genes in SCB35.

SCB34 and SCB58 were also MDR strains despite not being ESBL producers. In addition, 9 isolates (21%) were resistant to ciprofloxacin, and 16 (37%) were resistant to trimethoprim/sulfamethoxazole. No isolate was resistant to amikacin or to carbapenems.

### Phylogroup and MLST classification

The phylogroup and MLST classification of the neonatal *E*. *coli* strains is also included in Table 3. The majority of the isolates belonged to phylogroup B2 (n = 35, 81%). Five (12%) were classified in phylogroup D. One isolate each belonged to phylogroups A, B1, and F, respectively.

The most frequent sequence types overall were ST95 (n = 11, 25%), and ST131 (n = 9, 21%). SCB38, the only isolate associated with meningitis in our collection, was classified as ST95. The next most common ST was ST1193 (n = 3, 7%). ST95 and ST131 strains were present throughout the years studied, while ST1193 was only seen in isolates collected in recent years. ST95 and ST131 were found each in 17.6% of the isolates from newborns ≤ 72 h old. On the other hand, isolates from newborns > 72 h old belonged to ST95 in 30% of cases, and to ST131 in 23% of newborns at this age.

As shown in Table 3 ST95, ST131, and ST1193 isolates belong to phylogroup B2; other relevant STs within this phylogroup included ST73 (n = 2), ST127 (n = 2). Phylogroup D encompassed ST69 (n = 2), ST405 (n = 2), and ST501 (n = 1) strains. Correspondence of ST with respective phylogroup was confirmed according to data by Clermont et al. [13], and databases available at EnteroBase (www.enterobase.warwick.ac.uk).

### Prevalence of virulence factors

The presence of the 12 VFs we sought by PCR and of the K1 capsule determined by antigen detection is shown in the S3 Table. Sequencing of amplified PCR products detected in isolates SCB41, SCB42, SCB57, and SCB59 confirmed the presence of these virulence genes in these isolates, thus supporting the accuracy of our PCR methods (S5 table). All *E*. *coli* isolates carried *fimH*, *nlpI*, and *ompA*. The prevalence of the remaining VFs was as follows: *cnf1*, 35%; *hek/hra*, 35%; *hlyC*, 28%; *ibeA*, 16%; *iucC*, 58%; *iroN* 44%, *kpsMII*, 88%; *papGII-III*, 53%; *sfa/focDE*, 21%; and K1 capsule, 46% (Table 4). The median number of VFs in the 43 isolates was 7 (IQR 6–9). Isolates within phylogroup B2 or D had a significantly greater number of VFs compared to isolates within the other phylogroups (p<0.01). Regarding the distribution of VFs among the most common STs, namely ST95 and ST131, we found a significantly greater prevalence of *papGII-II* (p<0.001) and K1 (p<0.001) in ST95 isolates as compared to ST131 strains.

**Table 4:** Prevalence of virulence factors (VFs) in neonatal *Escherichia coli* bacteremia isolates, and VF frequency among isolates with ≥ 10 VFs compared to those with < 10 VFs.

	Total isolates N (%)	Isolates with ≥ 10 VFs N (%)	Isolates with <10 VFs N (%)	P value
*cnf1*	15 (35)	8 (100)	7 (20)	p < .001
*fimH*	43 (100)	8 (100)	35 (100)	NS
*hek/hra*	15 (35)	7 (87)	8 (23)	p < .01
*hlyC*	12 (28)	8 (100)	4 (11)	p < .001
*ibeA*	7 (16)	3 (37)	4 (11)	NS
*iroN*	19 (44)	7 (87)	12 (34)	p < .04
*iucC*	25 (58)	2 (25)	23 (66)	NS
*kpsMII*	38 (88)	7 (87)	31 (88)	NS
*nlpI*	43 (100)	8 (100)	35 (100)	NS
*ompA*	43 (100)	8 (100)	35 (100)	NS
*papGII-III*	23 (53)	8 (100)	15 (43)	NS
*sfa/foc DE*	9 (21)	7 (87)	2 (6)	p < .001
K1 capsule	20 (46)	4 (57)	18 (50)	NS

*cnf1* indicates cytotoxic necrotizing factor 1; *fimH*, type I fimbriae; *hek/hra*, adhesin/hemagglutinin; *hlyC*, hemolysin; *ibeA*, invasion of brain endothelium A; *iucC*, aerobactin; *iroN*, salmochelin; *kpsMT II*, capsule synthesis; *nlpI*, new lipoprotein I; *ompA*, outer membrane protein A; *papGII-III*, P fimbriae; *sfa/foc*, S fimbriae.

Table 4 also shows that *cnf1*, *hek/hra*, *hlyC*, *iroN*, and *sfa/focDE* were significantly more prevalent in isolates carrying ≥10 VFs compared to isolates with <10 VFs.

The number of VFs in isolates from newborns with bacteremia diagnosed at <72 hours of age was significantly greater than in the isolates from newborns diagnosed at ≥ 72 hours of age (mean±SD: 8.1±2.4 and 6.8±1.9, respectively; p<0.04). Among all the VFs tested, only *hek/hra* was significantly more prevalent in isolates from newborns who died compared to those from survivors (p<0.02).

We found an inverse correlation between the total number of VFs and the number of antibiotics to which the isolates were nonsusceptible (*r* = -0.47, p < .002).

## Discussion

This study was designed to prospectively identify newborns with *E*. *coli* bacteremia diagnosed in recent years in this US tertiary center. Our study demonstrates that the overall incidence of neonatal *E*. *coli* bacteremia at our institution surpasses the national estimates of neonatal invasive disease caused by GBS, the most common neonatal pathogen in the US (≤ 0.25 cases per 1,000 births) [27]. This is likely to be related to the predominance of preterm infants in our study, and to the continued decrease in early-onset GBS sepsis cases in the US in recent years [2].

In addition to characterizing the clinical information in newborns with *E*. *coli* bacteremia, we also sought to describe antibiotic resistance and to expand the knowledge of the molecular traits of this large collection of neonatal *E*. *coli* isolates. We found that most *E*. *coli* bacteremia occurred in premature neonates, and that the majority were diagnosed in the first 7 days of life. These findings are not unexpected, since *E*. *coli* is the pathogen that most frequently causes early-onset sepsis (EOS) in preterm infants [28]. The overall 28% mortality we observed is also consistent with the case fatality rate of 24 to 38% reported in other studies [28–30]. Prematurity is a well-recognized risk factor for the development of neonatal bacteremia [31], however, the specific conditions that increase the risk of *E*. *coli* bacteremia in US newborns have not been clearly defined. Multicenter case-control studies are needed to provide this information that is essential for designing preventive and treatment strategies against *E*. *coli* neonatal bacteremia.

We observed an ampicillin resistance rate of 67%, comparable to previous US studies of neonatal *E*. *coli* strains (rates between 64 and 85%) [2, 28–30, 32, 33]. Ampicillin resistance in neonatal *E*. *coli* bacteremia isolates has increased in this country since the implementation of group B *Streptococcus* intrapartum antibiotic prophylaxis, particularly in preterm newborns [32]. Whether the ampicillin resistance rates will continue to increase is unclear. The *E*. *coli* isolates we identified in the latter period of our study (years 2014–2016) did not demonstrate greater ampicillin rates compared to previous years. Interestingly, another recent study reported lower ampicillin resistance rates at 48% [34]. Taken together, these data highlight the need for continued vigilance of ampicillin resistance trends in invasive *E*. *coli* strains affecting newborns.

Few recent reports in the US include data on aminoglycoside resistance in neonatal *E*. *coli* bacteremia isolates. Gentamicin resistance has been reported to be between 0% and 10% [2, 28, 30, 32, 35]. The gentamicin resistance rate we observed is greater than the rates reported thus far in the US. This finding could be explained by differing regional antibiotic resistance patterns limited to the Oklahoma area, or by an overall increasing rate of resistance to gentamicin in invasive *E*. *coli* strains in the general US population, a rate that now surpasses 10% [36]. The increasing gentamicin resistance concomitant to ampicillin resistance in neonatal *E*. *coli* isolates observed by us and others [2, 32] is worrisome, particularly when considering that ampicillin plus gentamicin is the most common empiric antibiotic regimen used for neonates. Only one *E*. *coli* isolate, which was recovered in recent years, was resistant to 3^rd^ generation cephalosporins, similar to sporadic cephalosporin resistance findings from the limited data available in the US [28, 30]. ESBL-producing *E*. *coli* have been uncommonly reported [11, 28, 34]. One such isolate was detected among our neonatal *E*. *coli* bacteremia isolates at the end of 2013, but none were found thereafter.

Local susceptibility patterns of *E*. *coli* to available antibiotics need to be considered when deciding the most appropriate empiric regimen for neonatal bacteremia. Guidelines for empiric therapy of neonatal sepsis recommend the use of ampicillin plus gentamicin as the usual aminoglycoside of choice [37]. However, this regimen may not be the most appropriate in areas with high resistance to these antibiotics, particularly when simultaneous resistance to ampicillin and gentamicin is known to occur in neonatal *E*. *coli* bacteremia isolates [2, 3]. In a newborn with suspected meningitis, the use of cefotaxime or cefepime may be more appropriate until invasive Gram-negative infection is excluded. For locations with high rate (≥10%) of ESBL-producing E. coli, and meningitis is suspected, empiric therapy with meropenem is preferred over cephalosporins [38]

In addition to antibiotic resistance data, our study also provides relevant information on the genotypic characteristics of neonatal *E*. *coli* bacteremia strains. Our initial classification of neonatal *E*. *coli* bacteremia isolates used multi-locus sequence typing (MLST) [14]. Additional MLST characterization presented herein demonstrates that ST95 and ST131 continued to predominate overall among neonatal *E*. *coli* bacteremia strains over time. Interestingly, we also observed that ST95 and ST131 were found more frequently in newborns diagnosed > 72 h old as compared to infants diagnosed ≤ 72 h old. Although these differences did not reach statistical significance, it is tempting to hypothesize that these STs may be related to nosocomial transmission in newborns with prolonged length of stay after birth. Additional studies including techniques such as repetitive element sequence-based PCR or pulsed-field gel electrophoresis will likely aid to answer this question.

In addition to the predominance of ST95 and ST131 in our strains, we also identified novel invasive ST types such as ST1193 and ST127 have emerged among the newborn population. ST1193 strains have recently derived from ST14 [39] and, in contrast to most other ST14 members, commonly show resistance to fluoroquinolones [40] as we also demonstrated in the isolates of our collection. ST127 strains have been found in recent years as cause of bacteremia in older children and adults [41], and have been found to be highly virulent in experimental models of invasive *E*. *coli* infection [42]. A study of neonatal *E*. *coli* bacteremia isolates from France included one case by an ST127 strain [43]. Although an ST1193 strain has been reported as the cause of a single lethal case of neonatal meningitis [44], our study demonstrates for the first time the prevalence of ST1193 and ST127 among neonatal bacteremia isolates in the US.

Phylogroup assignment of *E*. *coli* is relevant because virulent extra-intestinal strains belong mainly to group B2 and, to a lesser extent, to group D, whereas commensal strains generally belong to group A [45]. There are very few data regarding the phylogroup classification of neonatal *E*. *coli* bacteremia isolated in the US. A recent US study of 28 newborns with *E*. *coli* bacteremia from 2006 to 2009 showed that most of the strains belonged to phylogroup B2 (68%), followed by phylogroup D (18%) [11]. Similarly, we also demonstrated a predominance of phylogroups B2 and D. Our study showed the presence of a phylogroup F (ST117) strain, which can be classified also within phylogroup B2 [13].This finding could be explained by our use of an updated phylogrouping method, which assigns phylogroups more accurately than the previous method [16]. Alternatively, the differing prevalence of non-B2 phylogroups in neonatal *E*. *coli* isolates could depend on regional factors. The predominance of phylogroups B2 and D in neonatal *E*. *coli* bacteremia isolates has been seen in other countries [43, 46–49], confirming the relevance of these phylogroups in defining their increased virulence properties [12].

Data are very limited on the prevalence of VFs in neonatal *E*. *coli* bacteremia strains. Our study demonstrates that, similar to invasive isolates in older populations, the neonatal B2 and D strains among our isolates had a significantly greater number of VFs compared to other *E*. *coli* phylogroups. The specific *E*. *coli* VFs that are relevant in the pathogenesis of neonatal sepsis are not well defined, partly due the variability among those reported in the few studies available to date. Our selection of VFs to be tested was based on previous studies in newborns, and also on available data of their known role in invasive infections in older hosts. Interestingly, we found that isolates from newborns diagnosed within 72 hours of life carried a significantly greater number of VFs compared to isolates from older newborns. The mechanisms and risk factors of EOS vs. late-onset sepsis (LOS) differ in part because EOS pathogens are vertically transmitted from a colonized mother to her newborn, whereas the sources of LOS organisms are more diverse. We hypothesize that some of the VFs that we tested could be responsible, at least partly, for the invasiveness of isolates transmitted perinatally from mother to infant in EOS. Alternatively, even if not directly involved, these VFs may be surrogates for other, yet unidentified VFs relevant to this pathogenic process. We found that *cnf1*, *hek/hra*, *hlyC*, *iroN*, and *sfa/focDE* were specifically more prevalent in strains with a greater number of VFs. Among this group, *hek/hra* was more prevalent in strains from newborns who died. The Hek adhesin mediates autoaggregation, adherence, and invasion of intestinal epithelial cells [50], and thus may also have a direct effect on pathogenesis of *E*. *coli* neonatal bacteremia. An isolate carrying *hek/hra* had specific liver tropism in an animal with lethal systemic invasive *E*. *coli* disease. These data may indicate that specific VFs including *hra/hek* determine severe outcomes of systemic infection [51].

The most prevalent VFs in our study were *fimH*, *nlpI*, *ompA*, and *kpsMII*. FimH, Nlp1, and OmpA are relevant to the pathogenesis of neonatal meningitis by promoting adhesion and invasion of human brain microvascular endothelial cells [52]. Therefore, it is possible they also play a role in early steps in the pathogenesis of neonatal bacteremia, which likely involve the bacterial adhesion to and invasion of epithelial barriers.

Group 2 capsules such as K1, K2, K5, and K15 are encoded by *kpsMII*. Group 2 capsules predominate in ExPEC isolates and are associated with virulence in animal models [53]. The well-characterized K1 capsule is present in up to 80% of *E*. *coli* isolates causing neonatal meningitis [54]. However, our study demonstrates that other group 2 capsules may be relevant to the pathogenesis of bacteremia in newborns, since the K1 was expressed in only 46% of strains in our collection. Interestingly, *ibeA*, which is relevant to the pathogenesis of newborn meningitis, was present in only 16% of our strains and was absent in the only isolate that was associated to meningitis in our study (SCB38). Its low prevalence in neonatal *E*. *coli* bacteremia isolates has been reported in other countries [43, 48, 55, 56]. These findings suggest that the VFs relevant to the pathogenesis of neonatal meningitis may be different from those that determine the development of the initial bacteremia event.

We observed that the presence of each *papGII-III* and K1 was significantly more frequent in ST95 strains compared to ST131 strains, the two most common STs in our collection. Another study showed that neonatal isolates belonging to ST95 (n = 6) or ST131 (n = 2) carried both pap adhesins and the K1 capsule[11]. Because of the limited number of neonatal *E*. *coli* bacteremia isolates reported in the US, larger studies will be needed to accurately determine the VF composition of these isolates in relation to their clonal characteristics.

We found an inverse correlation between VF content and antibiotic resistance traits in the neonatal strains we studied. Other authors have reported similar findings in human ExPECs, but not in animal isolates [57]. Further studies will be needed to clarify the relationship between virulence and antibiotic resistance in neonatal *E*. *coli* strains.

There are certain limitations to our study. The data represent newborns with *E*. *coli* bacteremia from only a single center. Unique local factors may have influenced the clinical characteristics, the antibiotic resistance, and the molecular epidemiology traits of the *E*. *coli* isolates that we investigated. Similar studies should be performed at other sites to determine the generalizability of our findings. In addition, we focused only on traditional VFs that are projected to be important in other ExPEC infections, but they may not necessarily be those involved in the pathogenesis of neonatal *E*. *coli* sepsis. As new information becomes available on the specific VFs relevant to neonatal *E*. *coli* bacteremia, studies on the prevalence of such traits will be needed. These additional studies will likely require the use of whole-genome sequencing (WGS) comparisons for a more detailed genetic characterization. We have generated WGS data from isolates collected in the earlier years of our study and have confirmed the prevalence of various virulence factors in these strains, but more comprehensive analyses encompassing larger number of neonatal *E*. *coli* isolates are needed [58] [59]. These studies are relevant because differences in specific molecular traits among neonatal *E*. *coli* strains likely determine at least in part the virulence phenotype of these isolates, as we have shown in our studies *in vitro* and in animal models [60]. Despite the limitations of our study, we believe that the data provide new and relevant information on contemporary resistance rates to several antibiotics in *E*. *coli* bacteremia isolates from US newborns. Moreover, our study provides a comprehensive molecular characterization of a large collection of *E*. *coli* bacteremia isolates from newborns in the US.

## Conclusion

We have characterized in detail the antibiotic resistance profile and several relevant molecular traits of a large collection of *E*. *coli* bacteremia isolates from US newborns. We found two thirds to be resistant to ampicillin, with simultaneous resistance to aminoglycosides in one of seven isolates. While ampicillin resistance did not increase over time, the overall gentamicin resistance rate we found was greater compared to other reports in the US. Resistance to other antibiotics was also common, and we found an ESBL isolate. These results are clinically relevant because they demonstrate that the treatment options for *E*. *coli* neonatal bacteremia are becoming increasingly limited.

We also showed that most isolates remain associated to phylogenetic group B2, and thus harbored several virulence genes typically found in ExPECs. The presence of these virulence genes may predict clinical outcomes, as we showed with the association of *hek/hra* with mortality in newborns. The role of additional VFs prevalent in neonatal *E*. *coli* bacteremia isolates in the pathogenesis and clinical outcomes of neonatal bacteremia needs to be investigated. This knowledge is also important because these factors can be used for molecular detection of virulent strains and could also be targets for vaccine development.

Continued surveillance of the resistance patterns and the molecular epidemiology of neonatal *E*. *coli* septicemia isolates is needed. Further definition of these characteristics in neonatal *E*. *coli* will also provide crucial novel information on relevant niches and modes of transmission of these invasive strains to newborns.

## Supporting information

S1 Table Former designation of *Escherichia coli* neonatal isolates with the corresponding nomenclature used in the present report. (DOCX)

S2 Table Risks factors and additional relevant clinical conditions in newborns with *E*. *coli* bacteremia. (DOCX)

S3 Table Presence (+) and absence (-) of individual virulence factors tested in neonatal *Escherichia coli* bacteremia isolates. (DOCX)

S4 Table Presence of selected beta-lactamase genes in isolate SCB35. (DOCX)

S5 Table Sequence confirmation and accession numbers of virulence factors in selected neonatal *E*. *coli* bacteremia isolates. (DOCX)
