# Supplementary material for: Antibiotic resistance and molecular characterization of bacteremia Escherichia coli isolates from newborns in the United States
Source: PLoS One. 2019 Jul 5;14(7):e0219352. doi: 10.1371/journal.pone.0219352 (PMC6611611; doi:10.1371/journal.pone.0219352)
Supplement: S1 Table — (DOCX) [file pone.0219352.s001.docx]

**S1 Table.** Former designation of *Escherichia coli* neonatal isolates with the corresponding nomenclature used in the present report.

| **Previous patient designation in Shakir et al. [14]** | ***E. coli* isolate designation in this manuscript** |
| --- | --- |
| Patient 1 | SCB 11 |
| Patient 2 | SCB 14 |
| Patient 3 | SCB 4 |
| Patient 4 | SCB 12 |
| Patient 5 | SCB 5 |
| Patient 6 | SCB 9 |
| Patient 7 | SCB 17 |
| Patient 8 | SCB 13 |
| Patient 9 | SCB 15 |
| Patient 10 | SCB 34 |
| Patient 11 | SCB 32 |
| Patient 12 | SCB 23 |
| Patient 13 | SCB 21 |
| Patient 14 | SCB 20 |
| Patient 15 | SCB 28 |
| Patient 16 | SCB 31 |
| Patient 17 | SCB 29 |
| Patient 18 | SCB 22 |
| Patient 19 | SCB 18 |
| Patient 20 | SCB 24 |
| Patient 21 | SCB 27 |
| Patient 22 | SCB 30 |
| Patient 23 | SCB 33 |
| Patient 24 | SCB 19 |
