# Supplementary material for: Antibiotic resistance and molecular characterization of bacteremia Escherichia coli isolates from newborns in the United States
Source: PLoS One. 2019 Jul 5;14(7):e0219352. doi: 10.1371/journal.pone.0219352 (PMC6611611; doi:10.1371/journal.pone.0219352)
Supplement: S2 Table — (DOCX) [file pone.0219352.s002.docx]

**S2 Table.** Risks factors and additional relevant clinical conditions in newborns with *E. coli* bacteremia.

| **Isolate** | **Chorio** | **Mec** | **5 m APGAR ≤ 6** | **ET** | **CVC** | **Congenital anomalies** | **Other conditions** |
| --- | --- | --- | --- | --- | --- | --- | --- |
| SCB 04 | no | no | no | yes | yes | none | no |
| SCB 05 | yes | no | no | yes | yes | none | no |
| SCB 09 | yes | no | no | no | yes | none | no |
| SCB 11 | yes | no | yes | yes | yes | none | no |
| SCB 12 | yes | no | yes | yes | no | ASD, inguinal hernia | no |
| SCB 13 | no | yes | no | no | no | none | IUGR, IDM |
| SCB 14 | yes | no | no | yes | yes | none | no |
| SCB 15 | no | no | no | no | no | none | LGA |
| SCB 17 | no | no | no | yes | yes | Malrotation, CDD, ASD | Pulmonary hemorrhage |
| SCB 18 | yes | no | no | no | no | none | no |
| SCB 19 | no | no | no | no | no | none | no |
| SCB 20 | yes | no | yes | yes | yes | Polydactyly | no |
| SCB 21 | yes | no | yes | no | no | none | no |
| SCB 22 | yes | no | yes | yes | no | none | Twin birth |
| SCB 23 | no | no | no | yes | yes | none | no |
| SCB 24 | yes | no | no | no | yes | none | no |
| SCB 27 | yes | yes | no | no | no | VUR | no |
| SCB 28 | no | yes | yes | no | no | none | no |
| SCB 29 | yes | yes | no | no | yes | none | no |
| SCB 30 | no | no | no | no | no | Trisomy 21, VSD | no |
| SCB 31 | yes | yes | yes | yes | no | none | no |
| SCB 32 | no | no | yes | yes | yes | Ear anomaly, micropenis | no |
| SCB 33 | yes | yes | no | no | no | none | IUGR |
| SCB 34 | yes | no | yes | yes | yes | none | no |
| SCB 35 | yes | no | no | yes | no | none | no |
| SCB 37 | yes | yes | no | no | no | none | Cardiac arrythmia |
| SCB 38 | no | no | no | no | no | Abnormal kidneys | Anemia, hydronephrosis |
| SCB 40 | yes | no | no | yes | no | none | no |
| SCB 41 | no | no | no | no | no | Hydrocephalus | no |
| SCB 42 | no | yes | no | no | yes | none | SGA |
| SCB 43 | no | no | n/r | no | no | VUR | no |
| SCB 45 | no | no | no | no | no | none | Infected cephalohematoma |
| SCB 47 | yes | no | no | no | no | none | no |
| SCB 49 | yes | yes | yes | yes | yes | none | no |
| SCB 50 | no | no | n/r | no | no | none | no |
| SCB 52 | no | no | no | no | no | none | no |
| SCB 54 | no | no | n/r | no | no | none | UTI |
| SCB 55 | no | no | no | no | yes | VSD, DORV | no |
| SCB 56 | yes | no | yes | yes | yes | none | no |
| SCB 57 | yes | no | no | no | no | none | no |
| SCB 58 | no | no | no | no | yes | RHH | no |
| SCB 59 | yes | no | no | yes | no | none | Pulmonary hemorrhage |
| SCB 60 | no | yes | yes | no | yes | none | Twin birth |

ASD indicates atrial septal defect; CDD, congenital diaphragmatic hernia; Chorio, history of maternal chorioamnionitis; CVC, presence of a central venous catheter; DORV, double-outlet right ventricle; ET, endotracheal intubation; IDM, infant of diabetic mother; IUGR, intrauterine growth retardation; LGA, large for gestational age; Mec, meconium-stained amniotic fluid; RHH, right hypoplastic heart; SGA, small for gestational age; UTI, urinary tract infection; VSD, ventricular septal defect; VUR, vesico-ureteral reflux
