# Supplementary material for: Antibiotic resistance and molecular characterization of bacteremia Escherichia coli isolates from newborns in the United States
Source: PLoS One. 2019 Jul 5;14(7):e0219352. doi: 10.1371/journal.pone.0219352 (PMC6611611; doi:10.1371/journal.pone.0219352)
Supplement: S3 Table — (DOCX) [file pone.0219352.s003.docx]

**S3 Table.** Presence (+) and absence (-) of individual virulence factors tested in neonatal *Escherichia coli* bacteremia isolates.

| Isolate | *cnf1* | *hek/hra* | *hlyC* | *ibeA* | *iucC* | *iroN* | *kpsMII* | *papGII-III* | *sfa/focDE* | K1 |
| --- | --- | --- | --- | --- | --- | --- | --- | --- | --- | --- |
| SCB 04 | - | - | - | - | - | + | + | + | - | + |
| SCB 05 | - | + | - | - | + | - | + | + | - | - |
| SCB 09 | - | - | - | + | - | - | + | - | - | - |
| SCB 11 | + | + | + | + | - | + | + | + | + | + |
| SCB 12 | + | + | + | + | - | + | + | + | + | + |
| SCB 13 | - | + | - | - | + | + | + | - | - | + |
| SCB 14 | + | + | + | - | - | + | - | + | + | - |
| SCB 15 | + | - | + | + | - | + | + | + | - | + |
| SCB 17 | + | - | - | + | + | + | + | + | - | - |
| SCB 18 | + | - | - | - | + | + | + | - | - | - |
| SCB 19 | - | - | - | - | - | + | + | + | - | + |
| SCB 20 | - | - | - | - | + | - | + | - | - | - |
| SCB 21 | - | + | - | - | + | + | - | - | - | - |
| SCB 22 | - | - | - | - | + | - | + | - | - | - |
| SCB 23 | - | - | - | - | + | - | + | - | - | - |
| SCB 24 | - | - | - | - | - | + | + | + | - | + |
| SCB 27 | - | - | - | - | + | + | + | + | - | + |
| SCB 29 | - | - | - | - | + | - | + | + | - | + |
| SCB 30 | - | - | - | - | + | - | + | - | - | - |
| SCB 31 | - | - | - | - | + | - | + | - | - | + |
| SCB 32 | - | - | - | - | + | + | + | + | - | + |
| SCB 33 | - | - | - | - | + | - | + | - | - | - |
| SCB 34 | + | + | + | - | + | - | + | + | + | - |
| SCB 35 | - | + | - | - | + | - | + | - | - | - |
| SCB 37 | - | - | - | - | + | - | + | - | - | + |
| SCB 38 | - | - | - | - | + | - | + | + | - | + |
| SCB 40 | + | - | - | - | + | - | + | - | - | - |
| SCB 41 | + | + | + | - | + | + | + | + | + | - |
| SCB 42 | + | + | + | - | - | + | + | + | + | - |
| SCB 43 | - | - | - | - | - | - | + | + | - | + |
| SCB 45 | - | - | - | - | + | - | + | - | - | + |
| SCB 47 | + | + | + | - | - | + | + | - | + | - |
| SCB 49 | - | - | - | - | - | - | - | - | - | - |
| SCB 50 | - | + | - | - | - | - | + | - | - | - |
| SCB 52 | - | - | + | - | + | + | + | + | - | + |
| SCB 54 | - | - | - | - | - | - | - | - | - | - |
| SCB 55 | - | - | - | - | - | - | + | + | - | + |
| SCB 56 | + | + | + | - | - | - | + | + | - | - |
| SCB 57 | + | + | + | + | - | + | + | + | + | + |
| SCB 58 | + | - | - | + | + | - | + | - | - | - |
| SCB 59 | + | + | + | - | - | + | - | + | + | - |
| SCB 60 | - | - | - | - | + | - | + | - | - | + |

*cnf1* indicates cytotoxic necrotizing factor 1; *hek/hra*, adhesin/hemagglutinin; *hlyC*, hemolysin; *ibeA*, invasion of brain endothelium A; *iucC*, aerobactin; *iroN*, salmochelin; *kpsMT II*, capsule synthesis; *papGII-III*, P fimbriae; *sfa/foc*, S fimbriae; K1, K1 capsule. Not shown are the virulence factors that were found in all the isolates tested and included *fimH*, type I fimbriae; *nlpI*, new lipoprotein I; and *ompA*, outer membrane protein A.
