# Supplementary material for: Antibiotic resistance and molecular characterization of bacteremia Escherichia coli isolates from newborns in the United States
Source: PLoS One. 2019 Jul 5;14(7):e0219352. doi: 10.1371/journal.pone.0219352 (PMC6611611; doi:10.1371/journal.pone.0219352)
Supplement: S4 Table — (DOCX) [file pone.0219352.s004.docx]

**S4 Table.** Presence of selected beta-lactamase genes in isolate SCB35.

| **Gene** | **Sequence** | **Accession No.** |
| --- | --- | --- |
| *bla_CTX-M-15_* | GCGGCCGCGCTACAGTACAGCGATAACGTGGCGATGAATAAGCTGATTGCTCACGTTGGCGGCCCGGCTAGCGTCACCGCGTTCGCCCGACAGCTGGGAGACGAAACGTTCCGTCTCGACCGTACCGAGCCGACGTTAAACACCGCCATTCCGGGCGATCCGCGTGATACCACTTCACCTCGGGCAATGGCGCAAACTCTGCGGAATCTGACGCTGGGTAAAGCATTGGGCGACAGCCAACGGGCGCAGCTGGTGACATGGATGAAAGGCAATACCACCGGTGCAGCGAGCATTCAGGCTGGACTGCCTGCTTCCTGGGTTGTGGGGGATAAAACCGGCAGCGGTGGCTATGGCACCACCAACGATATCGCGGTGATCTGGCCAAAAGATCGTGCGCCGCTGATTCTGGTCACTTACTTCACCCAGCCTCAACCTAAGGCAGAAAGCCGTC | MN025268 |
| *bla_OXA-1_* | CTTTTGCAAATATTATCTACAGCAGCGCCAGTGCATCAACAGATATCTCTACTGTTGCATCTCCATTATTTGAAGGAACTGAAGGTTGTTTTTTACTTTACGATGCATCCACAAACGCTGAAATTGCTCAATTCAATAAAGCAAAGTGTGCAACGCAAATGGCACCAGATTCAACTTTCAAGATCGCATTATCACTTATGGCATTTGATGCGGAAATAATAGATCAGAAAACCATATTCAAATGGGATAAAACCCCCAAAGGAATGGAGATCTGGAACAGCAATCATACACCAAAGACGTGGATGCAATTTTCTGTTGTTTGGGTTTCGCAAGAAATAACCCAAAAAATTGGATTAAATAAAATCAAGAATTATCTCAAAGATTTTGATTATGGAAATCAAGACTTCTCTGGAGATAAAGAAAGAAACAACGGATTAACAGAAGCATGGCTCGAAAGTAGCTTAAAAATTTCACCAGAAGAACAAATTCAATTCCTGCGTAAAATTATTAATCACAATCTCCCAGTTAAAAACTCAGCCATAGAAAACACCATAGAGAACATGTATCTACAAGATCTGGATAATAGTACAAAACTGTATGGGAAAACTGGTGCAGGATTCACAGCAAATAGAACCTTACAAAACGGATGGTTTGAAGGGTTTATTATAAGCAAATCAGGACATAAATATGTTTTTGTGTCCGCACTTACAGGAAACTTGGGGTCGAATTTAACATCAAGCATAAAAGCCAAGAAAAA | MN025269 |

The presence of groups I-IV CTX-M beta-lactamases was sought using the following primers and conditions [1] [2]:

CTX-M group I:

CTXM1-F3 5’-GACGATGTCACTGGCTGAGC-3’

CTXM1-R2 5’-AGCCGCCGACGCTAATACA-3’

CTX-M group II:

TOHO1-2F 5’-GCGACCTGGTTAACTACAATCC-3’

TOHO1-1R 5’-CGGTAGTATTGCCCTTAAGCC-3’

CTX-M group III:

CTXM825F 5’-CGCTTTGCCATGTGCAGCACC-3’

CTXM825R 5’-GCTCAGTACGATCGAGCC-3’

CTX-M group IV:

CTXM914F 5’-GCTGGAGAAAAGCAGCGGAG-3’

CTXM914R 5’-GTAAGCTGACGCAACGTCTG-3’

Conditions: 25 cycles of denaturation at 94°C for 30 s, 57°C for 30 s for annealing, and 72°C for 30 s for extension, followed by final elongation at 72°C for 10 minutes.

Detection of *bla*_OXA-1_ was done with the following primers and conditions [3]:

Fwd 5’-ACACAATACATATCAACTTCGC-3’

Rev: 5’-AGTGTGTTTAGAATGGTGATC-3’

Conditions: 40 cycles of 1 min of denaturation at 94°C, 2 min of annealing at 55°C, 3 min of extension at 72°C, and 10 min at 72°C for final extension.

Detection of TEM beta-lactamases was performed with the following primers and conditions [4] [5]:

TemA1 5’-ATAAAATTCTTGAAGAC-3’

TemB1 5’-TTACCAATGCTTAATCA-3’

Conditions: 36 cycles of 30 s of denaturation at 94°C, 30 s of annealing at 42°C, and 60 s of extension at 72°C, with a final extension step at 72°C for 10 min

Detection of SHV beta- lactamases was done with the following primers and conditions [4] [6]:

SHV-F 5’-CACTCAAGGATGTATTGTG-3’

SHV-R 5’-TTAGCGTTGCCAGTGCTCG-3’

Conditions: 24 cycles of denaturation at 96°C for 15 s, primer annealing at 50°C for 15 s, and extension at 72°C for 10 min.

**References:**

1. Pitout JD, Hossain A, Hanson ND. Phenotypic and molecular detection of CTX-M-beta-lactamases produced by Escherichia coli and Klebsiella spp. J Clin Microbiol. 2004;42(12):5715-21. Epub 2004/12/08. doi: 10.1128/JCM.42.12.5715-5721.2004. PubMed PMID: 15583304; PubMed Central PMCID: PMCPMC535227.

2. Priyadharsini RI, Kavitha A, Rajan R, Mathavi S, Rajesh KR. Prevalence of bla (CTX M) extended spectrum beta lactamase gene in enterobacteriaceae from critical care patients. J Lab Physicians. 2011;3(2):80-3. doi: 10.4103/0974-2727.86838. PubMed PMID: 22219559; PubMed Central PMCID: PMCPMC3249722.

3. Chmelnitsky I, Carmeli Y, Leavitt A, Schwaber MJ, Navon-Venezia S. CTX-M-2 and a new CTX-M-39 enzyme are the major extended-spectrum beta-lactamases in multiple Escherichia coli clones isolated in Tel Aviv, Israel. Antimicrob Agents Chemother. 2005;49(11):4745-50. Epub 2005/10/28. doi: 10.1128/AAC.49.11.4745-4750.2005. PubMed PMID: 16251320; PubMed Central PMCID: PMCPMC1280129.

4. Leflon-Guibout V, Jurand C, Bonacorsi S, Espinasse F, Guelfi MC, Duportail F, et al. Emergence and spread of three clonally related virulent isolates of CTX-M-15-producing Escherichia coli with variable resistance to aminoglycosides and tetracycline in a French geriatric hospital. Antimicrob Agents Chemother. 2004;48(10):3736-42. doi: 10.1128/AAC.48.10.3736-3742.2004. PubMed PMID: 15388428; PubMed Central PMCID: PMCPMC521882.

5. Speldooren V, Heym B, Labia R, Nicolas-Chanoine MH. Discriminatory detection of inhibitor-resistant beta-lactamases in Escherichia coli by single-strand conformation polymorphism-PCR. Antimicrob Agents Chemother. 1998;42(4):879-84. PubMed PMID: 9559800; PubMed Central PMCID: PMCPMC105559.

6. Pitout JD, Thomson KS, Hanson ND, Ehrhardt AF, Moland ES, Sanders CC. beta-Lactamases responsible for resistance to expanded-spectrum cephalosporins in Klebsiella pneumoniae, Escherichia coli, and Proteus mirabilis isolates recovered in South Africa. Antimicrob Agents Chemother. 1998;42(6):1350-4. PubMed PMID: 9624474; PubMed Central PMCID: PMCPMC105602.
