# Supplementary material for: Antibiotic resistance and molecular characterization of bacteremia Escherichia coli isolates from newborns in the United States
Source: PLoS One. 2019 Jul 5;14(7):e0219352. doi: 10.1371/journal.pone.0219352 (PMC6611611; doi:10.1371/journal.pone.0219352)
Supplement: S5 Table — (DOCX) [file pone.0219352.s005.docx]

**S5 Table.** Sequence confirmation of virulence factors in selected neonatal *E. coli* bacteremia isolates

| **Strain** | **Gene** | **Sequence** | **Accession No.** |
| --- | --- | --- | --- |
| SCB41 | *cnf1* | TGCAATAGAAAATGATGGTAATTTTAATGAGTCTTACTTTCTGTATTCCAATAAGACACTTAGCAATAAAGATGTTTTTGATGCTATAGCTATTTCTGTTAAGAAACGCAGTTTCAGTGATGGTGATATCGTTATAAAAT  CAAACAGTGAAGCTCAACGAGACTATGCTCTGACTATACTCCAGACGATTTTATCAATGACCCCTATATTTGATATCGTAGTCCCGGAGGTATCTGTTCCGCTTGGACTGGGGATTATTACTTCCAGTATGGGGATCAGTTTTGATCAACTGATTAATGGTGATACTTATGAAGAACGTCGTTCTGCTATACCTGGTTTGGCGACAAATGCAGTACTGCTTGGTCTGTCTTTTGCAATTCCACTCTTGATTAGTAAGGCAGGAATAAACCAGGAGGTACTTAGCAGCGTTATAAATAATGAGGGCAGGACTCTGAATGA | MK941168 |
|  | *fimH* | CGGTGAGCAGTGCGGGGGGAGTGGCGATTAAAGCTGGCTCATTAATTGCCGTGCTTATTTTGCGACAGACCAACAACTATAACAGCGATGATTTCCAGTTTGTGTGGAATATTTACGCCAATAATGATGTGGTGGTGCCCACTGGCGGCTGTGATGCTTCTGCTCGTGATGTCACCGTTACTCTGCCGGACTACCCTGGTTCAGTGCCGATTCCTCTTACCGTTTATTGTGCGAAAAGCCAAAACCTGGGGTATTACCTATCCGGCACAACCGCAGATGCGGGCAACTCGATTTTCACCAATACCGCGTCGTTTTCACCCGCGCAGGGCGTCGGCGTACAGTTGACGCGCAACGGTACGATTATTCCAGCGAATAACACGGTATCGTTAGGAGCAGTAGGGACTTCGGCGGTAAGTCTGGGATTAACGGCAAATTACGCACGTACCGGAGGGCAGGTGACTGC | MK941169 |
|  | *hek* | ATGAGTACGGAAGCAGTGGTCGCGAATCGTTGTCACGTTCAGGCTCTGCTGACAACTTCGCATGGAGCCTTGGCGCGGGTGTCCGCTATGACGTAACCCCGGATATCGCTCTGGACCTCAGCTATCGCTATCTTGATGCAGGTGACAGCAGTGTGAGTTACAAGGACGAGTGGGGCGATAAATATAAGTCAGAAGTTGATGTTAAAAGTCATGACATCATGCTTGG | MK941170 |
|  | *hlyC* | CAGAAACTGGCCAGTCTCTTTGTTTGCAATAAATGTATTACCTGCAATACGGGCTAACCAATATGCTTTATTAACCCGGGATAATTACCCTGTTGCATATTGTAGTTGGGCTAATTTAAGTTTAGAAAATGAAATTAAATATCTTAATGATGTTACCTCATTAGTCGCAGAAGACTGGACTTCAGGTGATCGTAAATGGTTCATTGACTGGATTGCTCCTTTCGGGGATAACGGTGCCCTGTACAAATATATGCGAAAAAAATTCCCTGATGAACTATTCCGAGCCATCAGGGTGGATCCCAAAACTCATGTTGGTAAAGTATCAGAATTTCACGGAGGTAAAATTGATAAACAGTTAGCGAATAAAATTTTTAAACAATATCACCACGAGTTAATAACTGAAGTAAAAAACAAGATAGATTTCAATTTTTCATTAACAGGTTAAGAGGTAATTAAATGCCAACAATAACCACTGCACAAATTAAAAGCACA | MK941171 |
|  | *ibeA* | Not present | Not present |
|  | *iroN* | TTTTCCACTGGTGAGAGCGTCTGACTGGAATACCAATGCCACATGGATGATCACTTCGGAGCAAAAAGACACCGGTAATCCTCTGTCGGTCATCCCGAAATATACTATCAATAACTCGCTTAACTGGACCATCACCCAGGCGTTTTCTGCCAGCTTCAACTGGACGTTATATGGCAGACAAAAACCGCGTACTCATGCGGAAACCCGCAGTGAA | MK941172 |
|  | *iucC* | CTCTGTATCACCTGCTTTGCCGCTACGGTGTCGCGCTTATTGCACATGGACAAAATATAACTCTCGCCATGAAAGAGGGGGTTCCACAGCGTGTTCTGCTGAAAGACTTCCAGGGCGATATGCGACTGGTGAAAGAAGAGTTCCCCGAAATGGACTCTTTGCCTCAGGAGGTTCGTGATGTTACATCCCGCCTGAGTGCGGACTACTTAATCCATGATTTGCAGACGGGTA | MK941173 |
|  | *kps* | ATGCCAGACATCTCATTCCCGGTGTTTTTACTTAATGGCCTGATTCCCTTTTTTATCTTTAGCAGTATCAGCAATCGTTCTGTAGGTGCTATTGAAGCGAATCAGGGGCTGTTTAATTATCGACCAGTAAAACCCATCGATACGATCATTGCGCGTGCATTGCTTGAGACACTGATTTATGTTGCTGTTTATATATTGCTTATGCTTATTGTCTGGAT | MK941174 |
|  | *nlp1* | ATACTTCCTGGCGTAAAAGTGAAGTCCTCGCGGTACCATTGCAACCGACTTTACAGCAGGAAGTGATTCTGGCACGTATGGAACAGATCCTTGCCAGTCGGGCTTTAACCGATGACGAACGCGCACAGCTTTTATATGAGCGCGGAGTGTTGTATGATAGTCTCGGTCTGAGGGCATTAGCGCGTAACGATTTTTCGCAAGCGCTGGC | MK941175 |
|  | *ompA* | GCTCAGTAGTTGCTCCGGCTCCAGCTCCGGCACCGGAAGTACAGACCAAGCACTTCACTCTGAAGTCTGACGTTCTGTTCACCTTCAACAAAGCAACCCTGAAACCGGAAGGTCAGGCTGCTCTGGATCAGCTGTACAGCCAGCTGAGCAACCTGGATCCGAAAGACGGTTCCGTAGTTGTTCTGGGTTACACTGACCGCATCGGTTCTGACGCTTATAACCAGGCTCTGTCCGAGCGTCGTGCTCAGTCCGTTGTTGATTACCTGATCTCTAAAGGTATCCCGGCAGACAAAATCTCCGCACGTGGTATGGGCGAATCCAACCCGGTTACTGGCAACACCTGTGACAAA | MK941176 |
|  | *papGII* | TAGGTTCCCTGCTTTTTTATTTTTATCCCTGTCAGGCTGTAATGATGCTTTGGCTATCCAGAGTACAATGTTTTACTCGTTTAATGATAACATTTATCGTCCTCGACTTAGTGTTAAAGTAACCGATGTTATTCAAATTATAGTGGATATAAACTCTGCATCAAGTACGGCAACTTTAAGCTATGTGGACTGCAATGGATTTACATGGTCTCATGGTATTTACTGGTCTGAGTATTTTGCATGGCTGGTTGTTCCTAAACGTGTTTCCTATAATGGATATGATATATATCTTGAACTTCAGTCCAGAGGAAGTTTTTCACTTGATGCAGAAGATAATGATAATTACTATCTTACCAAGGGATTTGCATGGGATGAAGCAAACACATCTGGACGGACATGTTTCAATATCGGAGAAAAAAGAA | MK941177 |
|  | *sfa* | ATGAACTGCATATGCAGGCGAGGTATAAGGCCACACATTATCCCGTCACCGGGGGAAAGGCAAATGGACAGGTATGGTTTTCTCTGACCTATCTGTAACTGGCAGATATAACGCCATTTAATTAAGGCTGTTAATAACATGATGAAGCACATGCGTATATGGGCCGTTCTGGCATCATTTTTAGTCTTTTTTTATATTCCGCAGAGCTATGCCGGGGTTGCTCTGGGGGCCACCCGTGTGATTTACCCTGAAGGGCAAAAACAGGTACAACTGGCGGTAACAAATAATGATGATAAAAGTAGTTACCTTATTCAGTCATGGATTGAAAATGCTGAGGGAAAAAAGGATGCCCGGTTTGTAATTACTCCTCCGAA | MK941178 |
| SCB42 | *cnf1* | ATGATGGTAATTTTAATGAGTCTTACTTTCTGTATTCCAATAAGACACTTAGCAATAAAGATGTTTTTGATGCTATAGCTATTTCTGTTAAGAAACGCAGTTTCAGTGATGGTGATATCGTTATAAAATCAAACAGTGAA  GCTCAACGAGACTATGCTCTGACTATACTCCAGACGATTTTATCAATGACCCCTATATTTGATATCGTAGTCCCGGAGGTATCTGTTCCGCTTGGACTGGGGATTATTACTTCCAGTATGGGGATCAGTTTTGATCAACTGATTAATGGTGATACTTATGAAGAACGTCGTTCTGCTATACCTGGTTTGGCGACAAATGCAGTATTGCTTGGTCTGTCTTTTGCAATTCCACTCTTGATTAGTAAGGCAGGAATAAACCAGGAGGTACTTAGCAGCGTTATAAATAATGA | MK953688 |
|  | *fimH* | TACGTGCGTAATTTGCCGTTAATCCCAGACTTACCGCCGAAGTCCCTACTGCTCCTAACGATACCGTGTTATTCGCTGGAATAATCGTACCGTTGCGCGTCAACTGTACGCCGACGCCCTGCGCGGGTGAAAACGACGCGGTATTGGTGAAAATCGAGTTGCCCGCATCTGCGGTTGTGCCGGAGAGGTAATACCCCAGGTTTTGGCTTTTCGCACAATAAACGGTAAGAGGAATCGGCACTGAACCAGGGTAGTCCGGCAAAGTAACGGTGACATCACGAGCAGAAACATCACAGCCGCCAGTGGGCACCACCACATCATTATTGGCGTAAATATTCCACACAAACTGGAAATCATCGCTGTTATAGTTGTTGGTCTGTCGCAAAATAAGCACGGCAATTAATGAGCCAGCTTTAATCGCCACTCCCCCCGCACTGCTCACAG | MK990716 |
|  | *hek* | TACCTGGGATTATGAGTACGGAAGCAGTGGTCGCGAATCGTTGTCACGTTCAGGCTCTGCTGACAACTTCGCATGGAGCCTTGGCGCGGGTGTCCGCTATGACGTAACCCCGGATATCGCTCTGGACCTCAGCTATCGCTATCTTGATGCAGGTGACAGCAGTGTGAGTTACAAGGACGAGTGGGGCGATAAATATAAGTCAGAAGTTGATGTTAAAAGTCATGA | MK990717 |
|  | *hlyC* | ACAGAAACTGGCCAGTTTCTTTGTTTGCAATAAATGTATTACCTGCAATACGGGCTAACCAATATGCTTTATTAACCCGGGATAATTACCCTGTTGCATATTGTAGTTGGGCTAATTTAAGTTTAGAAAATGAAATTAAATATCTTAATGATGTTACCTCATTAGTCGCAGAAGACTGGACTTCAGGTGATCGTAAATGGTTCATTGACTGGATTGCTCCTTTCGGGGATAACGGTGCCCTGTACAAATATATGCGAAAAAAATTCCCTGATGAACTATTCCGAGCCATCAGGGTGGATCCCAAAACTCATGTTGGTAAAGTATCAGAATTTCACGGAGGTAAAATTGATAAACAGTTAGCGAATAAAATTTTTAAACAATATCACCACGAGTTAATAACTGAAGTAAAAAACAAGACAGATTTCAATTT | MK990718 |
|  | *ibeA* | Not present | Not present |
|  | *iroN* | GAAAGCTCTGGTGGACGGTATCGAAGCCAGTATGTCTTTCCCACTGGTGAAAGATCGTCTGAACTGGAATACCAATGCCACATGGATGATCACTTCGGAGCAAAAAGACACCGGTAATCCTCTGTCGGTCATCCCGAAATATACTATCAATAACTCGCTTAACTGGACCATCACCCAGGCGTTTTCTGCCAACGTCAACTGGACGTTATA | MK990719 |
|  | *iucC* | Not present | Not present |
|  | *kps* | TTACATTATGCACCGCACGATGCCAGACATCTCATTCCCGGTGTTTTTACTTAATGGCCTGATTCCCTTTTTTATCTTTAGCAGTATCAGCAATCGTTCTGTAGGTGCTATTGAAGCGAATCAGGGACTGTTTAATTATCGACCAGTAAAACCCATCGATACGATCATTGCGCGTGCATTGCTTGAGACACTGATTTACGTTGCTGTTTATATATTGCTTATGCTTATCGTCTGGATGA | MK990720 |
|  | *nlp1* | TAATGCCCTCAGACCGAGACTATCATACAACACTCCGCGCTCATATAAAAGCTGTGCGCGTTCGTCATCGGTTAAAGCCCGACTGGCAAGGATCTGTTCCATACGTGCCAGAATCACTTCCTGCTGTAAAGTCGGTTGCAATGGTACCGCGAGGACTTCACTTTTACGCCAGGAAGTATTACTGCATCCTGCAAGCGTAAGTGCTGTCGCAACGAA | MK990721 |
|  | *ompA* | CAGCTCCAGTAGTTGCTCCGGCTCCAGCTCCGGCACCGGAAGTACAGACCAAGCACTTCACTCTGAAGTCTGACGTTCTGTTCACCTTCAACAAAGCAACCCTGAAACCGGAAGGTCAGGCTGCTCTGGATCAGCTGTACAGCCAGCTGAGCAACCTGGATCCGAAAGACGGTTCCGTAGTTGTTCTGGGTTACACTGACCGCATCGGTTCTGACGCTTATAACCAGGCTCTGTCCGAGCGTCGTGCTCAGTCCGTTGTTGATTACCTGATCTCTAAAGGTATCCCGGCAGACAAAATCTCCGCACGTGGTATGGGCGAATCCAACCCGGTTACTGGCAACACCTGTGACAAAA | MK990722 |
|  | *papGII* | TCGGCTCCCTGCTTTTTTATTTTTATCCCTGTCAGGCTGTAATGATGCTCTGGCTGCAAACCAGAGTACAATGTTTTACTCGTTTAATGATAACATTTATCGTCCTCAACTTAGTGTTAAAGTAACCGATATTGTTCAATTCATAGTGGATATAAACTCCGCATCAAGTACGGCAACTTTAAGCTATGTGGCCTGCAATGGATTTACCTGGACTCATGGTCTTTACTGGTCTGAGTATTTTGCATGGCTGGTTGTTCCTAAACATGTTTCCTATAATGGATATAATATATATCTTGAACTTCAGTCCAAAGGAGGTTTTTCACTTGATGCAGAAGATAATGATAATTACTATCTTACCAAGGGATTTGCATGGGATGAAGTAAACTCATCTGGACGGGTATGTTTCGATATCGGAGAAAAAAGAAGTCTGGCATGGTCATTTGGTGGTGTTACCCTGAACGCCAGATTGCCTGTTGACCTTCCTAAGGGGGATTATACGTTTCCAGTTAAGTTCTTACGTGGCATTCAGCGTAATAATTATGATTATATTGGTGGACGCTACAAAATCCCTTCCTCGTTAATGAAAACATTTCCTTTTAATGGTACATTGAATTTCTCAATTAAAAATACCGGAGGATGCCGTCCTTCTGCACAGTCTCTGGAAATAAATCATGGTGATCTGTCGATTAATAGCGCTAATAATCATTATGCGGCTCAGACTCTTTCTGTGTGTTGCGAT | MK990723 |
|  | *sfa* | CGCAATGACTGCATATGCAGGCGAGGTATAAGGCCACACATTATCCCGTCACCGGGGGAAAGGCAAATGGACAGGTATGGTTTTCTCTGACCTATCTGTAACTGGCAGATATAATGCCATTTAATTAAGGCTGTTAATAACATGATGAAGCACATGCGTATATGGGCCGTTCTGGCATCATTTTTAGTCTTTTTTTATATTCCGCAGAGCTATGCCGGGGTTGCTCTGGGTGCCACCCGTGTGATTTACCCTGAAGGGCAAAAACAGGTACAACTGGCGGTAACAAATAATGATGATAAAAGTAGTTACCTTATTCAATCATGGATTGAAAATGTTGAAGGAAAAAAGGATGCCAGGTTTGTAATTACTCCTCCGAGA | MK990724 |
| SCB57 | *cnf1* | ATGCAATAGAAAATGATGGTAATTTTAATGAGTCTTACTTTCTGTATTCCAATAAGACACTTAGCAATAAAGATGTTTTTGATGCTATAGCTATTTCTGTTAAGAAACGCAGTTTCAGTGATGGTGATATCGTTATAAAATCAAACAGTGAAGCTCAACGAGACTATGCTCTGACTATACTCCAGACGATTTTATCAATGACCCCTATATTTGATATCGTAGTCCCGGAGGTATCTGTTCCGCTTGGACTGGGGATTATTACTTCCAGTATGGGGATCAGTTTTGATCAACTGATTAATGGTGATACTTATGAAGAACGTCGTTCTGCTATACCTGGTTTGGCGACAAATGCAGTATTGCTTGGTCTGTCTTTTGCAATTCCACTCTTGATTAGTAAGGCAGGAATAAACCAGGAGGTACTTAGCAGCGTTATAAATAATGAGGGCAGGACTCTGAATG | MN022626 |
|  | *fimH* | CGGTGAGCAGTGCGGGGGGAGTGGCGATTAAAGCTGGCTCATTAATTGCCGTGCTTATTTTGCGACAGACCAACAACTATAACAGCGATGATTTCCAGTTTGTGTGGAATATTTACGCCAATAATGATGTGGTGGTGCCCACTGGCGGCTGCGATGTTTCTGCTCGTGATGTCACCGTTACTCTGCCGGACTACCCTGGTTCAGTGCCGATTCCTCTTACCGTTTATTGTGCGAAAAGCCAAAACCTGGGGTATTACCTCTCCGGCACAACCGCAGATGCGGGCAACTCGATTTTCACCAATACCGCGTCGTTTTCACCCGCGCAGGGCGTCGGCGTACAGTTGACGCGCAACGGTACGATTATTCCAGCGAATAACACGGTATCGTTAGGAGCAGTAGGGACTTCGGCGGTAAGTCTGGGATTAACGGCAAATTACGCACGTACCGGAGGGCAGGTGACTGCA | MN022627 |
|  | *hek* | AGCATGAGTACGGAAGCAGTGGTCGCGAATCGTTGTCACGTTCAGGCTCTGCTGACAACTTCGCATGGAGCCTTGGCGCGGGTGTCCGCTATGACGTAACCCCGGATATCGCTCTGGACCTCAGCTATCGCTATCTTGATGCAGGTGACAGCAGTGTGAGTTACAAGGACGAGTGGGGCGATAAATATAAGTCAGAAGTTGATGTTAAAAGTCATGACATCATGCTTGGTA | MN004968 |
|  | *hlyC* | ACGTTCCCCATTACACAGAAACTGGCCAGTTTCTTTGTTTGCAATAAATGTATTACCTGCAATACGGGCTAACCAATATGCTTTATTAACCCGGGATAATTACCCTGTTGCATATTGTAGTTGGGCTAATTTAAGTTTAGAAAATGAAATTAAATATCTTAATGATGTTACCTCATTAGTCGCAGAAGACTGGACTTCAGGTGATCGTAAATGGTTCATTGACTGGATTGCTCCTTTCGGGGATAACGGTGCCCTGTACAAATATATGCGAAAAAAATTCCCTGATGAACTATTCCGAGCCATCAGGGTGGATCCCAAAACTCATGTTGGTAAAGTATCAGAATTTCACGGAGGTAAAATTGATAAACAGTTAGCGAATAAAATTTTTAAACAATATCACCACGAGTTAATAACTGAAGTAAAAAACAAGACAGATTTCAATTTTTCATTAACAGGTTAAGAGG | MN022628 |
|  | *ibeA* | CCGCCGTTGATGTTATCAAGCAGGGCAATAATTTACTCGGCGTAATAACAGAGAGTAAATCTGGTCGTCAGGCTATTTTGGCAAATGTCATTATTGACTGTACTGGTGATGCTGATATTGCATGGTTTGCCGGAGCACCATTTATTAAGCGTGAACGCGAAGAGCTAATGTGTATGACAACCGTTTTTAGTTGCGCAAATATAAATAAAAA | MN022629 |
|  | *iroN* | TGTCTTTCCCGCTGGTGAAAGATCGTCTGAACTGGAATACCAATGCCACATGGATGATCACTTCGGAGCAAAAAGACACCGGTAATCCTCTGTCGGTCATCCCGAAATATACTATCAATAACTCGCTTAACTGGACCATCACCCAGGC | MN022630 |
|  | *iucC* | Not present | Not present |
|  | *kps* | AGGCGTTATGCACCGCACTATGCCAGACATCTCGTTCCCGGTGTTTTTACTTAATGGCCTGATTCCCTTTTTTATCTTTAGTAGTATTAGCAAACGTTCTATTGGTGCTATTGAAGCGAACCAGGGACTGTTTAATTATCGACCAGTAAAACCCATCGATACGATCATTGCACGTGCACTGCTTGAGACACTGATTTACGTTGCTGTTTATATTTTGCTCATGCTTATCGTCTGGATGA | MN022631 |
|  | *nlp1* | AGGATGCAGTAATACTTCCTGGCGTAAAAGTGAAGTTCTCGCGGTACCATTGCAACCGACTTTACAGCAGGAAGTGATTCTGGCACGTATGGAACAGATCCTTGCCAGTCGGGCTTTAACCGATGACGAACGCGCACAGCTTTTATATGAGCGCGGAGTGTTGTATGATAGTCTCGGTCTGAGGGCATTAGCGCGTAACGATTTTT | MN022632 |
|  | *opmA* | CMTTCGACAGCTCCAGTAGTTGCTCCGGCTCCAGCTCCGGCACCGGAAGTACAGACCAAGCACTTCACTCTGAAGTCTGACGTTCTGTTCACCTTCAACAAAGCAACCCTGAAACCGGAAGGTCAGGCTGCTCTGGATCAGCTGTACAGCCAGCTGAGCAACCTGGATCCGAAAGACGGTTCCGTAGTTGTTCTGGGTTACACTGACCGCATCGGTTCTGACGCTTATAACCAGGCTCTGTCCGAGCGTCGTGCTCAGTCCGTTGTTGATTACCTGATCTCTAAAGGTATCCCGGCAGACAAAATCTCCGCACGTGGTATGGGCGAATCCAACCCGGTTACTGGCAACACCTGTGACAAAA | MN022633 |
|  | *papGII* | AAGCTCGTGCTTTTTTATTTTTATCCCTGTCAGGCTGTAATGATGCTCTGGCTGCAAACCAGAGTACAATGTTTTACTCGTTTAATGATAACATTTATCGTCCTCAACTTAGTGTTAAAGTAACCGATATTGTTCAATTCATAGTGGATATAAACTCCGCATCAAGTACGGCAACTTTAAGCTATGTGGCCTGCAATGGATTTACCTGGACTCATGGTCTTTACTGGTCTGAGTATTTTGCATGGCTGGTTGTTCCTAAACATGTTTCCTATAATGGATATAATATATATCTTGAACTTCAGTCCAGAGGAAGTTTTTCACTTGATGCAGAAGATAATGATAATTACTATCTTACCAAGGGATTTGCATGGGATGAAGTAAACTCATCTGGACGGGTATGTTTCGATATCGGAGAAAAAAGAAGTCTGGCATGGTCATTTGGTGGTGTTACCCTGAACGCCAGATTGCCTGTTGACCTTCCTAAGGGGGATTATACGTTTCCAGTTAAGTTCTTACGTGGCATTCAGCGTAATAATTATGATTATATTGGTGGACGCTACAAAATCCCTTCCTCGTTAATGAAAACATTTCCTTTTAATGGTACATTGAATTTCTCAATTAAAAATACCGGAGGATGCCGTCCTTCTGCACAGTCTCTGGAAATAAATCATGGTGATCTGTCGATTAATAGCGCTAATAATCATTATGCGGCTCAGACTCTTTCTGTGTCTTGCGATGTGCCTACAAATATTC | MN022634 |
|  | *sfa* | CGCATGAAACTGCATATGCAGGCGAGGTATAAGGCCACACATTATCCCGTCACCGGGGGAAAGGCAAATGGACAGGTATGGTTTTCTCTGACCTATCTGTAACTGGCAGATATAATGCCATTTAATTAAGGCTGTTAATAACATGATGAAGCACATGCGTATATGGGCCGTTCTGGCATCATTTTTAGTCTTTTTTTATATTCCGCAGAGCTATGCCGGGGTTGCTCTGGGTGCCACCCGTGTGATTTACCCTGAAGGGCAAAAACAGGTACAACTGGCGGTAACAAATAATGATGATAAAAGTAGTTACCTTATTCAATCATGGATTGAAAATGTTGAAGGAAAAAAGGATGCCAGGTTTGTAATTACTCCTCCGAGA | MN022635 |
| SCB59 | *cnf1* | TCAYTMTGCAATAGAAAATGATGGTAATTTTAATGAGTCTTACTTTCTGTATTCCAATAAGACACTTAGCAATAAAGATGTTTTTGATGCTATAGCTATTTCTGTTAAGAAACGCAGTTTCAGTGATGGTGATATCGTTATAAAATCAAACAGTGAAGCTCAACGAGACTATGCTCTGACTATACTCCAGACGATTTTATCAATGACCCCTATATTTGATATCGTAGTCCCGGAGGTATCTGTTCCGCTTGGACTGGGGATTATTACTTCCAGTATGGGGATCAGTTTTGATCAACTGATTAATGGTGATACTTATGAAGAACGTCGTTCTGCTATACCTGGTTTGGCGACAAATGCAGTATTGCTTGGTCTGTCTTTTGCAATTCCACTCTTGATTAGTAAGGCAGGAATAAACCAGGAGGTACTTAGCAGCGTTATAAATAATGAATGAGAGAGCYCTGAAGGG | MK990798 |
|  | *fimH* | GGGGKGCTATTGMGCTGTGAGCAGTGCGGGGGGAGTGGCGATTAAAGCAGGCTCATTAATTGCCGTGCTTATTTTGCGACAGACCAACAACTATAACAGGGATGATTTCCAGTTTGTGTGGAATATTTACGCCAATAATGATGTGGTGGTGCCCACTGGCGGCTGCGATGTTTCTGCTCGTGATGTCACCGTTACTCTGCCGGACTACCCTGGTTCAGTGCCGATTCCTCTTACCGTTTATTGTGCGAAAAGCCAAAACCTGGGGTATTACCTCTCCGGCACAACCGCAGATGCGGGCAACTCGATTTTCACCAATACCGCGTCGTTTTCACCCGCGCAGGGCGTCGGCGTACAGTTGACGCGCAACGGTACGATTATTCCAGCGAATAACACGGTATCGTTAGGAGCAGTAGGGACTTCGGCGGTAAGTCTGGGATTAACGGCAAATTACGCACGTACCGGAGGGCAGGTGACTGC | MK990799 |
|  | *hek* | CAGNGCGCGAATCGTTGTCACGTTCAGGCTCTGCTGACAACTTCGCATGGAGCCTTGGCGCGGGTGTCCGCTATGACGTAACCCCGGATATCGCTCTGGACCTCAGCTATCGCTATCTTGATGCAGGTGACAGCAGTGTGAGTTACAAGGACGAGTGGGGCGATAAATATAAGTCAGAAGTTGATGTTAAAAGTCATGACATCATGCTTGGTATGACTTATAAC | MK990800 |
|  | *hlyC* | ATCATTACACAGAAACTGGCCAGTTTCTTTGTTTGCAATAAATGTATTACCTGCAATACGGGCTAACCAATATGCTTTATTAACCCGGGATAATTACCCTGTTGCATATTGTAGTTGGGCTAATTTAAGTTTAGAAAATGAAATTAAATATCTTAATGATGTTACCTCATTAGTCGCAGAAGACTGGACTTCAGGTGATCGTAAATGGTTCATTGACTGGATTGCTCCTTTCGGGGATAACGGTGCCCTGTACAAATATATGCGAAAAAAATTCCCTGATGAACTATTCCGAGCCATCAGGGTGGATCCCAAAACTCATGTTGGTAAAGTATCAGAATTTCACGGAGGTAAAATTGATAAACAGTTAGCGAATAAAATTTTTAAACAATATCACCACGAGTTAATAACTGAAGTAAAAAACAAGACAGATTTCAA | MK990801 |
|  | *ibeA* | Not present | Not present |
|  | *iroN* | CAKTYCTGGTGGACGGTATCGAAGCCAGTATGTCTTTCCCGCTGGTGAAAGATCGTCTGAACTGGAATACCAATGCCACATGGATGATCACTTCGGAGCAAAAAGACACCGGTAATCCTCTGTCGGTCATCCCGAAATATACTATCAATAACTCGCTTAACTGGACCATCACCCAGGCGTTTTCTGCCAGCT TCAACTGGACGTTATATGGCA | MK990802 |
|  | *iucC* | Not present | Not present |
|  | *kps* | Not present | Not present |
|  | *nlp1* | CSAGCACTTACGCTTGCAGGATGCAGTAATACTTCCTGGCGTAAAAGTGAAGTCCTCGCGGTACCATTGCAACCGACTTTACAGCAGGAAGTGATTCTGGCACGTATGGAACAAATCCTTGCCAGTCGGGCTTTAACCGATGACGAACGCGCACAGCTTTTATATGAGCGCGGAGTGTTGTATGATAGTCTCGGTCTGAGGGCATTAG | MK990803 |
|  | *opmA* | CMTTCGACAGCTCCAGTAGTTGCTCCGGCTCCAGCTCCGGCACCGGAAGTACAGACCAAGCACTTCACTCTGAAGTCTGACGTTCTGTTCACCTTCAACAAAGCAACCCTGAAACCGGAAGGTCAGGCTGCTCTGGATCAGCTGTACAGCCAGCTGAGCAACCTGGATCCGAAAGACGGTTCCGTAGTTGTTCTGGGTTACACTGACCGCATCGGTTCTGACGCTTATAACCAGGCTCTGTCCGAGCGTCGTGCTCAGTCCGTTGTTGATTACCTGATCTCTAAAGGTATCCCGGCAGACAAAATCTCCGCACGTGGTATGGGCGAATCCAACCCGGTTACTGGCAACACCTGTGACAAAA | MK990804 |
|  | *papGII* | TTTTTATTTTTATCCCTGTCAGGCTGTAATGATGCTCTGGCTGCAAACCAGAGTACAATGTTTTACTCGTTTAATGATAACATTTATCGTCCTCAACTTAGTGTTAAAGTAACCGATATTGTTCAATTCATAGTGGATATAAACTCCGCATCAAGTACGGCAACTTTAAGCTATGTGGCCTGCAATGGATTTACCTGGACTCATGGTCTTTACTGGTCTGAGTATTTTGCATGGCTGGTTGTTCCTAAACATGTTTCCTATAATGGATATAATATATATCTTGAACTTCAGTCCAGAGGAAGTTTTTCACTTGATGCAGAAGATAATGATAATTACTATCTTACCAAGGGATTTGCATGGGATGAAGTAAACTCATCTGGACGGGTATGTTTCGATATCGGAGAAAAAAGAAGTCTGGCATGGTCATTTGGTGGTGTTACCCTGAACGCCAGATTGCCTGTTGACCTTCCTAAGGGGGATTATACGTTTCCAGTTAAGTTCTTACGTGGCATTCAGCGTAATAATTATGATTATATTGGTGGACGCTACAAAATCCCTTCCTCGTTAATGAAAACATTTCCTTTTAATGGTACATTGAATTTCTCAATTAAAAATACCGGAGGATGCCGTCCTTCTGCACAGTCTCTGGAAATAAATCATGGTGATCTGTCGATTAATAGCGCTAATAATCATTATGCGGCTCAGACTCTTTCTGTGTCTTGCGATGTGCCTACAAATATTCGTTTTTTCCTGTTAAGCAATACAACTCCGGCATACAGCCATGGTCAGCAATTTTCGGTTGGTCTGGGTCATGGCTGGGACTCCATTGTTTCGATTAATGGCGTGGACACAGGAGAGACAACGATGAGATGGTACAGAGCAGGTACACAAAACCTGACCAT | MK990805 |
|  | *sfa* | CGGATGACTGCATATGCAGGCAAGGTATAAGGCCACACATTATCCCGTCACCGGGGGAAAGGCAAATGGACAGGTATGGTTTTCTCTGACCTATCTGTAACTGGCAGATATAACGCCATTTAATTAAGGCTGTTAATAACATGATGAAGCACATGCGTATATGGGCCGTTCTGGCATCATTTTTAGTCTTTTTTTATATTCCGCAGAGCTATGCCGGGGTTGCTCTGGGTGCCACCCGTGTGATTTACCCTGAAGGGCAAAAACAGGTACAACTGGCGGTAACAAATAATGATGATAAAAGTAGTTACCTTATTCAGTCATGGATTGAAAATGCTGAAGGAAAAAAGGATGCCAGGTTTGTAATTACTCCTCCGATA | MK990806 |
